# Supplementary material for: Diagnosing and defining MASLD in people living with chronic hepatitis B
Source: Commun Med (Lond). 2026 May 8;6:273. doi: 10.1038/s43856-026-01383-2 (PMC13156279; doi:10.1038/s43856-026-01383-2)
Supplement: Supplementary file 2 — Supplementary material [file 43856_2026_1383_MOESM2_ESM.pdf]

SUPPLEMENT

Diagnosing and defining MASLD in chronic hepatitis B: a narrative review of non-invasive tests

CONTENTS

**Acronyms and abbreviations**		**P2**
**Suppl Table 1:**	Evidence gaps in appraisal of cardiometabolic factors used to define MASLD, which may influence integrity of estimates in populations with high prevalence of chronic hepatitis B (CHB) virus infection.	**P3**
**Suppl Table 2:**	Histological scoring systems used in Steatotic Liver Disease (SLD)	**P4-6**
**Suppl Table 3:**	Summary of Non-Invasive Tests (NITs) and their performance to diagnose Steatotic Liver Disease (SLD) in chronic hepatitis B (CHB) infection.	**P7-10**
**Suppl Table 4:**	Summary of Non-Invasive Tests (NITs) and their performance to diagnose hepatic fibrosis in chronic hepatitis B (CHB) infection.	**P11-20**
**Suppl Table 5:**	MASLD-specific risk stratification scores in chronic HBV infection (i.e. detection of MASH or "at-risk MASH" (MASH + fibrosis))	**P21-23**
**References**		**P24-28**

Acronyms and abbreviations (used consistently throughout this document)

- ALT - alanine transaminase
- AST - aspartate aminotransaminase
- AUROC - area under the receiver operator curve
- BMI - Body Mass Index
- CHB - Chronic hepatitis B infection
- CI - Confidence Interval
- FLD - Fatty Liver Disease Index
- GGT - Gamma Glutamyl Transferase
- HbA1c - Haemoglobin A1c (glycosylated haemoglobin)
- HBV - hepatitis B virus
- HCV - hepatitis C virus
- HSI - hepatic steatosis index
- HDL - high density lipoprotein
- LDL - low density lipoprotein
- NAFLD - Non–Alcoholic Fatty Liver Disease (terminology used prior to change to MASLD in 2023)
- MASH - Metabolic Dysfunction-Associated Steatohepatitis
- MASLD - Metabolic Dysfunction-Associated Steatotic Liver Disease
- PLWHB - People Living with Hepatitis B
- SIHBV - Steatosis Index of HBV infection
- TDF - Tenofovir Disoproxil Fumarate

Suppl Table 1: Evidence gaps in appraisal of cardiometabolic factors used to define MASLD, which may influence integrity of estimates in populations with high prevalence of chronic hepatitis B (CHB) virus infection .

**Factor**	**Caveats in populations with high HBV prevalence**
Serum lipid profile	● Profiles in black African populations differ from European populations which may affect MASLD diagnosis in different parts of the world[1].<br>● HBV is also reported to affect serum triglycerides, although this interaction is not fully understood[2].<br>● There is evidence to suggest that TDF (first line nucleot(s)ide analogue treatment in PLWHB) reduces total cholesterol and LDL, but also in HDL, compared with those not taking antiviral therapy, and people without HBV infection[3].
Diabetes mellitus	● HbA1c measurement is influenced by haemoglobinopathies (e.g. sickle cell disease, thalassaemia) which are more common in some regions of high HBV prevalence[4].
Overweight / Obesity	● Cardiovascular and mortality risks are associated with a lower BMI in individuals with Asian/African ancestry compared to White-European or American populations[5,6].<br>● Waist circumference measures central adiposity and is better correlated with risk but its use is still limited in clinical practice and measurement is not standardised[7].
Hypertension	● Cut-offs are largely arbitrary and risk of adverse events increases progressively above ~115/75 mmHg[8].<br>● Adverse outcomes may also vary according to ethnicity, and some studies suggest ethnicity-specific hypertension thresholds[9].

Suppl Table 2: Histological scoring systems used in steatotic liver disease[10–12]. Only scoring systems referred to in this review are included (not an exhaustive list).

**Liver pathology**	**Score name**	**Score categories**	**Notes**
Fibrosis	Metavir	Five-point scale:<br>● F0 = no fibrosis<br>● F1 = portal fibrosis without septa (mild fibrosis)<br>● F2 = few septa (significant fibrosis)<br>● F3 = numerous septa without cirrhosis (advanced fibrosis)<br>● F4 = cirrhosis	Also has a necroinflammatory scoring component.
	Ishak	Six stage scoring system.<br>● 0 = no fibrosis<br>● 1-2 = mild/moderate fibrosis (~ corresponds to Metavir F1)<br>● 3 = moderate fibrosis (~ corresponds to metavir F2)<br>● 4-5 = severe fibrosis (~corresponds to metavir F3)<br>● 6 = cirrhosis	Most detailed system.<br>Also has a necroinflammatory scoring component.
	Scheuer	Simple scoring system.<br>● 0 = no fibrosis<br>● 1 = Enlarged fibrotic portal tracts<br>● 2 = Periportal or portal=portal septa but intact architecture<br>● 3 = fibrosis with architectural distortion but no obvious cirrhosis<br>● cirrhosis	Studies usually define what they consider as advanced fibrosis in their methods. E.g. S2 significant fibrosis, S3 advanced fibrosis[13,14]<br>Also has a necroinflammatory scoring component.

Liver pathology	Score name	Score categories	Notes
MASH + fibrosis	Brunt	MASH severity scoring system. Three components:<br>● Steatosis grade<br>0. None<br>1. ≤ 33% steatosis<br>2. 33-66% steatosis<br>3. ≥66% steatosis<br>● Degree of lobular inflammation<br>0. None<br>1. Mild<br>2. Moderate<br>3. Marked<br>● Fibrosis<br>0. None<br>1. Perisinusoidal fibrosis<br>2. Periportal and perisinusoidal fibrosis<br>3. Bridging fibrosis<br>4. Cirrhosis	Studies define what they consider as advanced fibrosis in their methods e.g. Advanced fibrosis = Brunt fibrosis stage ≥ 3[15].

Liver pathology	Score name	Score categories	Notes
MASH	NAFLD Activity Score (NAS)	Used to grade activity of MASH. Grades three histological features:<br>● Steatosis:<br>0. <5%<br>1. 5-33%<br>2. 34-66%<br>3. >66%<br>● Lobular inflammation<br>0. None<br>1. 1-2 foci per 200x field<br>2. 3-4 foci per 200x field<br>3. >4 foci per 200x field<br>● Hepatocyte ballooning<br>0. None<br>1. Few<br>2. Many<br>Total of 8 points.<br>● 0-2 not diagnostic of MASH<br>● 3-4 indeterminate<br>● ≥ 5 MASH	Developed to grade rather than diagnose MASH, therefore can result in false positives.
“At-risk MASH”	● Often defined as NAS ≥4 + F2/3 fibrosis		Target group for clinical trials in MASLD

Supplementary Table 3: Summary of NITs and their performance to diagnose steatotic liver disease (SLD) in chronic hepatitis B (CHB) infection.

Test	Method / input data	Study	Population	Steatosis reference	Steatosis Grade	AUROC (95% CI)	Cut-off values	Sensitivity (%)	Specificity (%)
Ultrasound	Conventional B mode ultrasound	Herneaz, 2014[16]	N=4720 Mixed liver disease (study does not specify HBV)	Liver biopsy	n/a	0.93 (0.91-0.95)	n/a	84.8	93.6
Quantitative ultrasound	Controlled attenuation parameter	Karlas, 2017[17]	N=3830 (37% HBV = 1003)	Liver biopsy (S0 0-5%; S1 5-33%; S2-3 >33%)	S1	0.82 (0.81-0.84)	248 (237-261)	68.8	82.2
					S2	0.87 (0.85-0.88)	268 (257-284)	77.3	81.2
					S3	0.89 (0.86-0.91)	280 (268-294)	88.2	77.6
		Petroff, 2021[18]	N=474 (Viral hepatitis, HBV + HCV)	Liver biopsy (S0 0-5%;S1 5-33%;S2-3 >33%)	S1	0.77 (0.72-0.81)	230 (209-266)	71.4	68.0
					S2/3	0.85 0.79-0.90)	264 (238-285)	76.0	79.4

**Test**	**Method / input data**	**Study**	**Population**	**Steatosis reference**	**Steatosis Grade**	**AUROC (95% CI)**	**Cut-off values**	**Sensitivity (%)**	**Specificity (%)**
HSI	ALT, AST, BMI, sex, diabetes	Xu, 2017[19]	N= 366 treatment naive HBV (137 NAFLD); China	Liver biopsy (S0: <5%; S1: 5–33%; S2: 34–66%; S3: >66%)	S1	0.66 (0.60–0.70)	35.6	61	63
					S2	0.76 (0.71–0.80)	35.9	100	58.5
					S3	0.79 (0.74–0.83)	37.5	100	66
		Zhang, 2016[20]	N=364 HBV (182 model group, 182 validation group); China	Liver biopsy	n/a	0.63 (0.55-0.71) Validation group	n/a	n/a	n/a
		Chang, 2020[21]	N=244 HBV (45 SLD); South Korea	MRI-PDFF	n/a	0.73 (0.65- 0.81)	33.4	84.4	57.8

Test	Method / input data	Study	Population	Steatosis reference	Steatosis Grade	AUROC (95% CI)	Cut-off values	Sensitivity (%)	Specificity (%)
FLI	BMI, waist circumference, triglycerides, GGT	Zhang, 2016[20]	N=364 HBV (182 model group, 182 validation group); China	Liver biopsy	n/a	0.75 (0.67-0.83) Validation group	n/a	n/a	n/a
Korean Score	ALT, AST, GGT, triglycerides, BMI	Zhang, 2016[20]	N=364 HBV (182 model group, 182 validation group); China	Liver biopsy	n/a	0.65 (0.56-0.75) Validation group	n/a	n/a	n/a
LAP	Waist circumference, triglycerides	Zhang, 2016[20]	N=364 HBV (182 model group, 182 validation group); China	Liver biopsy	n/a	0.68 (0.57-0.78) Validation group	n/a	n/a	n/a
FLD	BMI, triglycerides, hyperglycaemia, AST, ALT	Zhang, 2016[20]	N=364 HBV (182 model group, 182 validation group); China	Liver biopsy	n/a	0.75 (0.75-0.82) Validation group	n/a	n/a	n/a

Test	Method / input data	Study	Population	Steatosis reference	Steatosis Grade	AUROC (95% CI)	Cut-off values	Sensitivity (%)	Specificity (%)
SIHBV	Haemoglobin, serum uric acid, age, triglycerides	Zhang, 2016[20]	N=364 HBV (182 model group, 182 validation group); China	Liver biopsy	n/a	0.86 (0.79-0.92) Validation group	Rule in ≥ 0.48	n/a	93.1
							Rule out <0.18	90.7	n/a

Supplementary Table 4: NITs and their performance to diagnose fibrosis in chronic hepatitis B (CHB) and metabolic dysfunction-associated steatotic liver disease (MASLD).

Reference test was liver biopsy in all studies. *cut-off derived via Youden's index.

Test	Method / input data	Study	Population	Advanced fibrosis reference	Liver disease in population tested	AUROC (95% CI)	Cut-off values	Sensitivity (95% CI)	Specificity (95% CI)
Fib-4	Age, AST, ALT, platelets	Xiao, 2014[22]	Meta-analysis, 6455 HBV	Liver biopsy (metavir /Batts/Ludvig/ Scheuer >2, Ishak > F3)	HBV	0.81 (SD, 0.03)	Rule in >3.25	17%	98%
							Rule out <1.45	63% (range 50-71%)	56% (range14-80%)
		Xiao, 2017[23]	Meta-analysis 13294 NAFLD	Liver fibrosis (Brunt & Kleiner, Metavir, Ludwig, or SAF scoring system > F2)	NAFLD	0.80 (0.77–0.84)	Rule in >3.25	37.3%	95.8%
							Rule out <1.30	77.8%	71.2%
		Luo, 2022[24]	China, 85 HBV/NAFLD	Transient elastography (>9.8kPa)	HBV/NAFLD	0.83	1.07	75%	80%

Test	Method / input data	Study	Population	Advanced fibrosis reference	Liver disease in population tested	AUROC (95% CI)	Cut-off values	Sensitivity (95% CI)	Specificity (95% CI)
		Lin, 2023[15]	Singapore + China; 2262 HBV, 984 HBV /SLD	Liver biopsy (ISHAK score ≥4 or Metavir score ≥3 or Brunt score ≥3)	HBV	0.63 (0.6-0.66)	Rule in >3.75	15.3% (12.2-18.8%)	94.4% (92.6-96.0%)
							Rule out <1.45	75.5% (70.9- 79.6%)	45.7% (42.3 - 49.1%)
					HBV/ SLD	0.60 (0.56-0.64)	Rule in >3.75	12.4% (9.0 - 16.4%)/	94.7% (92.7 - 96.3%)
							Rule out <1.45	82.2% (77.8 - 86.0%)	43.1% (39.0 - 47.1%)

Test	Method / input data	Study	Population	Advanced fibrosis reference	Liver disease in population tested	AUROC (95% CI)	Cut-off values	Sensitivity (95% CI)	Specificity (95% CI)
		Rui, 2024[13]	China; 88 HBV/SLD, 877 HBV/MASLD (1-3 CMF), 98 HBV/MASLD (4-5 CMF)	Liver biopsy (Scheuer classification ≥ 2)	HBV/SLD	0.78 (0.68-0.86)	1.26*	57.5%	87.8%
					HBV/MASLD (1-3 CMF)	0.65 (0.62-0.68)	1.26*	45.3%	79.0%
					HBV/MASLD (4-5 CMF)	0.63 (0.53-0.72)	0.8*	80.0%	43.8%
		Rui, 2024[14]	China; 794 HBV/MASLD	Liver biopsy (Scheuer classification ≥ 3)	HBV/MASLD	0.73 (0.69-0.78)	Rule in >2.09	n/a	90%
							Rule out <0.71	90%	n/a
		Li, 2017[25]	China; 131 HBV/NAFLD	Liver biopsy (Metavir score ≥ 3)	HBV/NAFLD	0.72 (0.70-0.80)	0.77	86%	36%
		Li, 2020[26]	China, 116 HBV/NAFLD	Liver biopsy (Metavir score ≥ 3)	HBV/NAFLD	0.68 (0.58-0.69)	n/a	n/a	n/a

Test	Method / input data	Study	Population	Advanced fibrosis reference	Liver disease in population tested	AUROC (95% CI)	Cut-off values	Sensitivity (95% CI)	Specificity (95% CI)
		Chen, 2024[27]	China, 844 HBV/SLD	Liver histology (Knodell histological activity index ≥ F3)	HBV/NAFLD	0.77 (0.74 - 0.80)	1.41	71%	78%
					HBV/MASLD	0.77 (0.73- 0.80)	1.41	69%	77%
					HBV/MAFLD	0.78 (0.75- 0..81)	1.41	75%	76%
					HBV/MetALD	0.77 (0.65- 0.86)	1.41	83%	71%
					Overall SLD	0.77 (0.74- 0.80)	1.41	73%	76%
APRI	AST, platelets	Xiao, 2015[22]	Meta-analysis, 8855 HBV	Liver biopsy (metavir /Batts/Ludvig/ Scheuer ≥2,Ishak ≥ F3	HBV	0.7407 (SD 0.0191)	Rule in >1.5	34.1% (range, 14.0-75.0%)	89.5% (range, 81.6-100.0%)
							Rule out <0.5	70.0% (range, 35.0-97.0)	60.0 (range, 34.0-86.7%)

Test	Method / input data	Study	Population	Advanced fibrosis reference	Liver disease in population tested	AUROC (95% CI)	Cut-off values	Sensitivity (95% CI)	Specificity (95% CI)
		Xiao, 2017	Meta-analysi, 13294 NAFLD	Liver fibrosis (Brunt & Kleiner, Metavir, Ludwig, or SAF scoring system ≥ F3)	NAFLD	0.75 (0.72–0.77)	Rule in >1.5	32.9%	90.5%
							Rule out <0.5	72.9%	67.7%
		Luo, 2022[24]	China, 85 HBV/NAFLD	Transient elastography (≥9.8kPa)	HBV/NAFLD	0.77	0.29	75%	67%
		Lin, 2023[15]	Singapore + China; 2262 HBV, 984 HBV /SLD	Liver biopsy (ISHAK score ≥4 or Metavir score ≥3 or Brunt score ≥3)	HBV	0.63 (0.60–0.66)	Rule in>1.5	29.2% (25.2-33.5%)/	81.2% (78.2%-83.9%)
							Rule out ≤0.5	55.7% (50.6-60.7%)	72.4% (69.3- 75.4%)
					HBV/ SLD	0.60 (0.56–0.64)	Rule in>1.5	19.7% (15.5-24.4%)	87.6% (84.8-90.1%)
							Rule out ≤0.5	58.2% (52.8-63.3%)	62.3% (58.3-66.2%)

Test	Method / input data	Study	Population	Advanced fibrosis reference	Liver disease in population tested	AUROC (95% CI)	Cut-off values	Sensitivity (95% CI)	Specificity (95% CI)
		Rui, 2024[13]	China; 88 HBV/SLD, 877HBV/MASLD (1-3 CMF), 98 HBV/MASLD (4-5 CMF)	Liver biopsy (Scheuer classification $\geq$ 2)	HBV/SLD	0.78 (0.68-0.86)	0.56*	66.0%	82.9%
					HBV/MASLD (1-3 CMF)	0.68 (0.64-0.71)	0.39*	69.2%	58.8%
					HBV/MASLD (4-5 CMF)	0.60 (0.49-0.69)	0.54*	50.0%	79.2%
		Rui, 2024[14]	China; 794 HBV/MASLD	Liver biopsy (Scheuer classification $\geq$ 3)	HBV/MASLD	0.71 (0.67-0.76)	n/a	n/a	n/a
		Li, 2017[25]	China; 131 HBV/NAFLD	Liver biopsy (Metavir score $\geq$ 3)	HBV/NAFLD	0.77 (0.68-0.84)	0.41*	80%	59%
		Li, 2020[26]	China, 116 HBV/NAFLD	Liver biopsy (Metavir score $\geq$ 3)	HBV/NAFLD	0.75 (0.66-0.82)	n/a	n/a	n/a

Test	Method / input data	Study	Population	Advanced fibrosis reference	Liver disease in population tested	AUROC (95% CI)	Cut-off values	Sensitivity (95% CI)	Specificity (95% CI)
		Chen, 2024[27]	China, 844 HBV/SLD	Liver histology (Knodell histological activity index ≥ F3)	HBV/NAFLD	0.74 (0.71-0.77)	0.39	79%	59%
					HBV/MASLD	0.74 (0.70-0.77)	0.39	78%	59%
					HBV/MAFLD	0.76 (0.72-0.79)	0.57	65%	75%
					HBV/MetALD	0.84 (0.73-0.92)	0.42	100%	61%
					Overall SLD	0.75	0.39	81%	58%
GPR	GGT, platelets	Lemoine, 2016[28]	West Africa, 135 HBV	Liver biopsy (Metavir score ≥ 2)	HBV	0.80 (0.72-0.88)	0.32	83%	69%
		Li, 2017[25]	China; 131 HBV/NAFLD	Liver biopsy (Metavir score ≥ 3)	HBV/NAFLD	0.89 (0.82-0.94)	0.49	83%	80%
		Li, 2020[26]	China, 116 HBV/NAFLD	Liver biopsy (Metavir score ≥ 3)	HBV/NAFLD	0.77 (0.68-0.84)	n/a	n/a	n/a

Test	Method / input data	Study	Population	Advanced fibrosis reference	Liver disease in population tested	AUROC (95% CI)	Cut-off values	Sensitivity (95% CI)	Specificity (95% CI)
		Luo, 2022[24]	China, 85 HBV/NAFLD	Transient elastography (≥9.8kPa)	HBV/NAFLD	0.81	0.31	75%	80%
		Chen, 2024[27]	China, 844 HBV/SLD	Liver histology (Knodell histological activity index ≥ F3)	HBV/NAFLD	0.75 (0.71-0.78)	0.38	66%	71%
					HBV/MASLD	0.74 (0.71-0.78)	0.31	74%	63%
					HBV/MAFLD	0.75	0.38	71%	69%
					HBV/MetALD	0.84 (0.74-0.92)	0.45	100%	65%
					Overall SLD	0.75 (0.72-0.78)	0.38	69%	69%

Test	Method / input data	Study	Population	Advanced fibrosis reference	Liver disease in population tested	AUROC (95% CI)	Cut-off values	Sensitivity (95% CI)	Specificity (95% CI)
Enhanced Liver Fibrosis (ELF)	3 serum markers of collagen metabolism (HA, PIIINP, TIMP-1)	Hinkson, 2023[29]	19,285, multi-site (meta-analysis) multiple liver aetiologies (10,417 NAFLD, 1120 HBV)	Liver biopsy (advance fibrosis: METAVIR, Batts-Ludqig, Kleiner/NASH CRN score 3, Ishak score 4 or Knodell score 2)	Multi-aetiology	0.81 (0.76–0.86)	9.8	72% (64 – 79%)	78% (71-83%)
AGILE 3+	LSM with AST/ALT ratio, platelet count, sex, diabetes status, and age	Papatheodoridi, 2024[30]	Multi-site (Europe); HBV 597, MASLD 912	Liver biopsy (Metavir $\geq$ 3)	MASLD	0.86 (0.84-0.89)	Rule in >0.68	n/a	86%
							Rule out <0.35	91%	n/a
					HBV	0.79 (0.75-0.83)	n/a	n/a	n/a

Test	Method / input data	Study	Population	Advanced fibrosis reference	Liver disease in population tested	AUROC (95% CI)	Cut-off values	Sensitivity (95% CI)	Specificity (95% CI)
		Rui, 2024[14]	China; 794 HBV/MASLD	Liver biopsy (Scheuer classification > 3)	HBV/MASLD	0.83 (0.80-0.87)	Rule in >0.720	n/a	90%
							Rule out <0.306	90%	n/a
LSM	Transient elastography by Fibroscan (Echosens)	Papatheodoridi, 2021[31]	Europe, 1073 NAFLD, 716 HBV	Liver fibrosis (Brunt & Kleiner, > F3)	HBV	n/a	Rule in >12	45%	94%
							Rule out <7	83%	66%
					NAFLD	0.87 (0.86–0.88)	Rule in >12	61%	88%
							Rule out <8	93%	49%
		Li, 2020[26]	China, 116 HBV/NAFLD	Liver biopsy (Metavir score > 2)	HBV/NAFLD	0.87 (0.79-0.92)	Rule in >10.8	n/a	90%
							Rule out <8	90%	n/a
		Rui, 2024[14]	China; 794 HBV/MASLD	Liver biopsy (Scheuer classification > 3)	HBV/MASLD	0.80 (0.75-0.85)	Rule in >11.9	n/a	90%
							Rule out <6.6	90%	n/a

Test	Method / input data	Study	Population	Advanced fibrosis reference	Liver disease in population tested	AUROC (95% CI)	Cut-off values	Sensitivity (95% CI)	Specificity (95% CI)
		Liu, 2022[32]	China, HBV 229, HBV/MAFLD 207	Liver biopsy (Metavir score $\geq$ 2)	HBV/MAFLD (none-mild steatosis CAP<268)	0.87 (0.83–0.91)	7.0	82%	86%
					HBV/MAFLD (mod- severe steatosis CAP $\geq$268)	0.83 (0.75–0.90)	8.8	90%	66%
		Shen, 2019[33]	China, HBV 370, HBV/NAFLD 223	Liver biopsy (Metavir score $\geq$ 2)	HBV/NAFLD (CAP <268)	0.80 (0.76-0.84)	8.8	64% (57-70%)	83% (77-86%)
					HBV/NAFLD (CAP $\geq$268)	0.74 (0.65-0.84)		72% (57-83%)	75% (64-85%)

Test	Method / input data	Study	Population	Advanced fibrosis reference	Liver disease in population tested	AUROC (95% CI)	Cut-off values	Sensitivity (95% CI)	Specificity (95% CI)
		Chen, 2024[27]	China, 844 HBV/SLD	Liver histology (Knodell histological activity index ≥ F3)	HBV/NAFLD	0.84 (0.81-0.87)	9	72%	81%
					HBV/MASLD	0.85 (0.82-9.87)	9	74%	81%
					HBV/MAFLD	0.86 (0.84-9.89)	8.7	81%	77%
					HBV/MetALD	0.79 (0.68-0.88)	8.8	83%	82%
					Overall SLD	0.84 (0.81-0.87)	9	74%	81%

*optimal cut off as per Youden's index

n/a – not available (parameter not available in manuscript)

Suppl Table 5: MASLD-specific risk stratification scores in chronic HBV infection i.e. detection of MASH, MASLD + fibrosis or "at-risk MASH" (MASH + fibrosis)

Test	Components	Study	Population	MASH reference	Condition	AUROC (95% CI)	Cut-off values	Sensitivity (95% CI)	Specificity (95% CI)
Serum cytokeratin -18	CK-18 M30	Tada, 2021,[34]	Japan, N=185 NASH, N=61 NAFL	Liver biopsy (Younossi criteria)	NASH	0.77	260*	83%	57%
	CK-18 M30	Dai, 2024[35]	China, N= 133 HBV/NASH	Liver biopsy (Scheur 2-3)	HBV/NASH + fibrosis	0.81 (0.63-0.90)	587.87*	83%	79%
	CK-18 M30/CK-18 M65	Dai, 2024[35]	China, N=133 HBV/NASH	Liver biopsy (Scheur 2-3)	HBV/NASH + fibrosis	0.84 (0.79-0.89)	483.49*	85%	80%
NFS	age, body mass index, blood glucose, platelet count, albumin, and aspartate aminotransferase [AST] to alanine aminotransferase [ALT] ratio	Xiao, 2017[23]	Meta-analysis NAFLD N=13294	Liver fibrosis (Brunt & Kleiner, Metavir, Ludwig, or SAF scoring system ≥ F2)	NASH + fibrosis	0.72 (0.65-0.79)	-1.1	66% (61-70%)	83% (69-96%)

Test	Components	Study	Population	MASH reference	Condition	AUROC (95% CI)	Cut-off values	Sensitivity (95% CI)	Specificity (95% CI)
		Rui, 2024[13]	China; 88 HBV/SLD, 877 HBV/MASLD (1-3 CMF), 98 HBV/MASLD (4-5 CMF)	Liver biopsy (Scheuer classification ≥ 2)	HBV/SLD + fibrosis	0.72 (0.62 - 0.81)	-3.55	83%	54%
					HBV/MASLD (1-3 CMF) +fibrosis	0.63 (0.6-0.67)	-2.15	54%	67%
					HBV/MASLD (4-5 CMF) + fibrosis	0.57 (0.46-0.67)	-1.00	48%	71%
		Li, 2020[26]	China, 116 HBV/NAFLD	Liver biopsy (Metavir score ≥ 3)	HBV/NAFLD + fibrosis	0.60 (0.50-0.69)	n/a	n/a	n/a
		Lin, 2023[15]	Singapore + China; 2262 HBV, 984 HBV /SLD	Liver biopsy (ISHAK score ≥4 or Metavir score ≥3 or Brunt score ≥3)	HBV/SLD + fibrosis	0.652 (0.61- 0.69)	Rule in >0.65	3% (1-6%)	99% (98 - 100%)
							Rule out < -1.455	83% (78-88%)	32% (28%-37%)

Test	Components	Study	Population	MASH reference	Condition	AUROC (95% CI)	Cut-off values	Sensitivity (95% CI)	Specificity (95% CI)
GBC Score[36]	AST, PT, PLT, ALB, GGT, HBeAg-positive, HBsAg, WBC, INR, BMI, and Tbil	Rui, 2024[36]	China; 1787 treatment-naive HBV/SLD (689 training set, 509 validation cohort 1, 589 validation cohort 2)	Liver biopsy (Scheur's criteria $\geq$ G3: moderate to severe inflammation)	HBV/MASH	0.89 (0.86-0.92) (validation cohort 1)	0.218	85%	79%
PPDHG[37]	Platelets, prothrombin time, diabetes, hepatitis B e Antigen, immunoglobulin	Wang, 2023	China, 504 treatment-naive HBV/NAFLD (336 training set, 168 validation set)	Liver biopsy (Scheur $\geq$ S3)	HBV/MASLD + fibrosis	0.82 (0.77-0.86)	-0.98	82%	67%

*cut-off derived via Youden's index. n/a - parameter not available in manuscript.

SUPPLEMENTARY REFERENCES

1. Greiner, R. *et al.* Associations between low HDL, sex and cardiovascular risk markers are substantially different in sub-Saharan Africa and the UK: analysis of four population studies. *BMJ Glob Health* **6**, (2021).
2. Wu, Y. *et al.* Small hepatitis B virus surface antigen (SHBs) induces dyslipidemia by suppressing apolipoprotein-AII expression through ER stress-mediated modulation of HNF4α and C/EBPγ. *J Virol* e0123924 (2024).
3. Tong, K. *et al.* Effects of first-line nucleot(s)ide analogues on lipid profiles in patients with chronic hepatitis B: a network meta-analysis. *Eur J Clin Pharmacol* **80**, 335–354 (2024).
4. Smaldone, A. Glycemic control and hemoglobinopathy: When A1C may not be reliable. *Diabetes Spectr.* **21**, 46–49 (2008).
5. Rao, G. *et al.* Identification of obesity and cardiovascular risk in ethnically and racially diverse populations: A scientific statement from the American heart association. *Circulation* **132**, 457–472 (2015).
6. Overview | Obesity: identification, assessment and management | Guidance | NICE.
7. Su, Y., Sun, J.-Y., Su, Z.-Y. & Sun, W. Revisiting waist circumference: A hypertension risk factor that requires a more in-depth understanding. *Curr. Cardiol. Rev.* **20**, e270324228382 (2024).
8. Mancia, G. *et al.* 2023 ESH Guidelines for the management of arterial hypertension The Task Force for the management of arterial hypertension of the European Society of Hypertension: Endorsed by the International Society of Hypertension (ISH) and the European Renal Association (ERA). *J Hypertens* **41**, 1874–2071 (2023).
9. Su, D. *et al.* Ethnicity-specific blood pressure thresholds based on cardiovascular and renal complications: a prospective study in the UK Biobank. *BMC Med.* **22**, 54 (2024).

10. Chowdhury, A. B. & Mehta, K. J. Liver biopsy for assessment of chronic liver diseases: a synopsis. *Clin Exp Med* **23**, 273–285 (2023).

11. UpToDate. https://www.uptodate.com/contents/histologic-scoring-systems-for-chronic-liver-disease.

12. Bell, P. Liver Pathology: Steatohepatitis. https://www.aasld.org/liver-fellow-network/core-series/pathology-pearls/liver-pathology-steatohepatitis.

13. Rui, F. *et al.* The Reduced Accuracy of Non-invasive Tests for Significant Fibrosis in Chronic Hepatitis B Patients with Metabolic Dysfunction-associated Steatotic Liver Disease. *J Clin Transl Hepatol* **12**, 827–829 (2024).

14. Rui, F., Ni, W., Shi, J., Xie, Q. & Li, J. Superior diagnostic efficacy of Agile 3+ score for diagnosing advanced fibrosis in patients with chronic hepatitis B and concurrent metabolic dysfunction-associated steatotic liver disease. *J Hepatol* (2024) doi:10.1016/j.jhep.2024.07.009.

15. Lin, K. W. *et al.* The utility of non-invasive tests to assess advanced fibrosis in Asian subjects with chronic hepatitis B and concomitant hepatic steatosis. *Liver Int* **43**, 1008–1014 (2023).

16. Hernaez, R. *et al.* Diagnostic accuracy and reliability of ultrasonography for the detection of fatty liver: a meta-analysis. *Hepatology* **54**, 1082–1090 (2011).

17. Karlas, T. *et al.* Individual patient data meta-analysis of controlled attenuation parameter (CAP) technology for assessing steatosis. *J Hepatol* **66**, 1022–1030 (2017).

18. Petroff, D. *et al.* Assessment of hepatic steatosis by controlled attenuation parameter using the M and XL probes: an individual patient data meta-analysis. *Lancet Gastroenterol Hepatol* **6**, 185–198 (2021).

19. Xu, L. *et al.* A comparison of hepatic steatosis index, controlled attenuation parameter and ultrasound as noninvasive diagnostic tools for steatosis in chronic hepatitis B. *Dig Liver Dis* **49**, 910–917 (2017).
20. Zhang, Z., Wang, G., Kang, K., Wu, G. & Wang, P. Diagnostic accuracy and clinical utility of a new noninvasive index for hepatic steatosis in patients with hepatitis B virus infection. *Sci Rep* **6**, 32875 (2016).
21. Chang, J. W. *et al.* Hepatic steatosis index in the detection of fatty liver in patients with chronic hepatitis B receiving antiviral therapy. *Gut Liver* **15**, 117–127 (2021).
22. Xiao, G., Yang, J. & Yan, L. Comparison of diagnostic accuracy of aspartate aminotransferase to platelet ratio index and fibrosis-4 index for detecting liver fibrosis in adult patients with chronic hepatitis B virus infection: a systemic review and meta-analysis. *Hepatology* **61**, 292–302 (2015).
23. Xiao, G. *et al.* Comparison of laboratory tests, ultrasound, or magnetic resonance elastography to detect fibrosis in patients with nonalcoholic fatty liver disease: A meta-analysis. *Hepatology* **66**, 1486–1501 (2017).
24. Luo, J. *et al.* Gamma-Glutamyl Transpeptidase-to-Platelet ratio predicts liver fibrosis in patients with concomitant chronic hepatitis B and nonalcoholic fatty liver disease. *J Clin Lab Anal* **36**, e24596 (2022).
25. Li, Q., Lu, C., Li, W., Huang, Y. & Chen, L. The gamma-glutamyl transpeptidase to platelet ratio for non-invasive assessment of liver fibrosis in patients with chronic hepatitis B and non-alcoholic fatty liver disease. *Oncotarget* **8**, 28641–28649 (2017).
26. Li, Q., Huang, C., Xu, W., Hu, Q. & Chen, L. Accuracy of FibroScan in analysis of liver fibrosis in patients with concomitant chronic Hepatitis B and nonalcoholic fatty liver disease. *Medicine (Baltimore)* **99**, e20616 (2020).
27. Chen, L. *et al.* Noninvasive tests maintain high accuracy for advanced fibrosis in chronic hepatitis B patients with different

nomenclatures of steatotic liver disease. *J Med Virol* **96**, e29613 (2024).

28. Lemoine, M. *et al.* The gamma-glutamyl transpeptidase to platelet ratio (GPR) predicts significant liver fibrosis and cirrhosis in patients with chronic HBV infection in West Africa. *Gut* **65**, 1369–1376 (2016).
29. Hinkson, A. *et al.* Meta-analysis: Enhanced liver fibrosis test to identify hepatic fibrosis in chronic liver diseases. *Aliment. Pharmacol. Ther.* **57**, 750–762 (2023).
30. Agile scores in MASLD and ALD: External validation and their utility in clinical algorithms. *Journal of Hepatology* **81**, 590–599 (2024).
31. Papatheodoridi, M. *et al.* Refining the Baveno VI elastography criteria for the definition of compensated advanced chronic liver disease. *J Hepatol* **74**, 1109–1116 (2021).
32. Liu, J. *et al.* Hepatic steatosis leads to overestimation of liver stiffness measurement in both chronic hepatitis B and metabolic-associated fatty liver disease patients. *Clin Res Hepatol Gastroenterol* **46**, 101957 (2022).
33. Shen, F. *et al.* Moderate to severe hepatic steatosis leads to overestimation of liver stiffness measurement in chronic hepatitis B patients without significant fibrosis. *Aliment Pharmacol Ther* **50**, 93–102 (2019).
34. Tada, T. *et al.* Predictive value of cytokeratin-18 fragment levels for diagnosing steatohepatitis in patients with nonalcoholic fatty liver disease. *Eur J Gastroenterol Hepatol* **33**, 1451–1458 (2021).
35. Dai, L., Yan, Y. & Chen, Q. Clinical significance of serum Ck18-M65 and M30 levels in patients with chronic hepatitis B combined with nonalcoholic steatohepatitis and liver fibrosis. *Medicine (Baltimore)* **103**, e38342 (2024).
36. Rui, F. *et al.* Development of a machine learning-based model to predict hepatic inflammation in chronic hepatitis B patients with

concurrent hepatic steatosis: a cohort study. *EClinicalMedicine* **68**, 102419 (2024).

37. Wang, J. *et al.* A novel non-invasive model for the prediction of advanced liver fibrosis in chronic hepatitis B patients with NAFLD. *J Viral Hepat* **30**, 287–296 (2023).
